# An artificial intelligence model for the pathological diagnosis of invasion depth and histologic grade in bladder cancer

**DOI:** 10.1186/s12967-023-03888-z

**Published:** 2023-01-23

**Authors:** Jiexin Pan, Guibin Hong, Hong Zeng, Chengxiao Liao, Huarun Li, Yuhui Yao, Qinghua Gan, Yun Wang, Shaoxu Wu, Tianxin Lin

**Affiliations:** 1 Department of Urology, Sun Yat-sen Memorial Hospital, Sun Yat-sen University, 107th Yanjiangxi Road, Guangzhou, China; 2 Guangdong Provincial Key Laboratory of Malignant Tumor Epigenetics and Gene Regulation, Sun Yat-sen Memorial Hospital, Sun Yat-sen University, Guangzhou, China; 3 Department of Pathology, Sun Yat-sen Memorial Hospital, Sun Yat-sen University, Guangzhou, China; 4 Guangdong Provincial Clinical Research Center for Urological Diseases, Guangzhou, Guangdong China

**Keywords:** Artificial intelligence, Bladder cancer, Pathological diagnosis, Muscle invasion, Histologic grade

## Abstract

**Background:**

Accurate pathological diagnosis of invasion depth and histologic grade is key for clinical management in patients with bladder cancer (BCa), but it is labour-intensive, experience-dependent and subject to interobserver variability. Here, we aimed to develop a pathological artificial intelligence diagnostic model (PAIDM) for BCa diagnosis.

**Methods:**

A total of 854 whole slide images (WSIs) from 692 patients were included and divided into training and validation sets. The PAIDM was developed using the training set based on the deep learning algorithm ScanNet, and the performance was verified at the patch level in validation set 1 and at the WSI level in validation set 2. An independent validation cohort (validation set 3) was employed to compare the PAIDM and pathologists. Model performance was evaluated using the area under the curve (AUC), accuracy, sensitivity, specificity, positive predictive value and negative predictive value.

**Results:**

The AUCs of the PAIDM were 0.878 (95% CI 0.875–0.881) at the patch level in validation set 1 and 0.870 (95% CI 0.805–0.923) at the WSI level in validation set 2. In comparing the PAIDM and pathologists, the PAIDM achieved an AUC of 0.847 (95% CI 0.779–0.905), which was non-inferior to the average diagnostic level of pathologists. There was high consistency between the model-predicted and manually annotated areas, improving the PAIDM’s interpretability.

**Conclusions:**

We reported an artificial intelligence-based diagnostic model for BCa that performed well in identifying invasion depth and histologic grade. Importantly, the PAIDM performed admirably in patch-level recognition, with a promising application for transurethral resection specimens.

**Supplementary Information:**

The online version contains supplementary material available at 10.1186/s12967-023-03888-z.

## Background

Bladder cancer (BCa) is the tenth most common cancer worldwide [1, 2]. According to the depth of tumour invasion, BCa can be divided into muscle-invasive BCa (MIBC) and non-muscle-invasive BCa (NMIBC). Patients with NMIBC are treated differently from those with MIBC. Transurethral resection of bladder tumour (TURBT) is the major strategy for treating NMIBC with postoperative infusion chemotherapy in medium–high risk patients to prevent tumour recurrence [3]. In contrast, radical cystectomy and pelvic lymph node dissection are required for MIBC, as well as adjuvant chemotherapy and immunotherapy when there is evidence of metastases [4]. In additional, tumour grade is one of the most important factor in predicting their biological aggressiveness [5, 6]. Recurrence and progression rates in high-grade patients are greater than in low-grade patients, suggesting that high-grade patients require closer long-term follow-up. In general, NMIBC has a favourable treatment outcome, with a five-year survival rate of up to 90% [7], whereas MIBC has a relatively poor prognosis, with a five-year survival rate of only 66% [8, 9]. Clearly, an accurate diagnosis of BCa is critical for clinical decision making and prognosis prediction.

Currently, noninvasive imaging examinations, including CT and MRI, are employed to aid in the preoperative diagnosis of BCa. In a previous study, we also proposed an MRI-based radiomic-clinical nomogram for the individualized preoperative prediction [10]. However, the diagnostic accuracy was less than satisfactory, with a reported range from 64.7 to 83% [11–14]. An erroneous diagnosis of MIBC or NMIBC might lead to undertreatment or overtreatment. Hence, diagnostic transurethral resection is still necessary to obtain postoperative specimens for definitive diagnosis and accurate tumour staging if imaging indicates a tumour-like lesion in the bladder.

Nevertheless, some dilemmas still remain regarding the pathological diagnosis. The identification of tissue specimens acquired following transurethral resection is challenging since they are frequently of poor quality, being fragmented, scarce or lacking a full muscle layer, resulting in potential misdiagnoses [15–17]. As is widely known, the process of pathological diagnosis is a labour-intensive, time-consuming and experience-dependent task. Depending on the complexity of cases, a thorough and comprehensive examination can take a few minutes to tens of minutes. For atypical cancerous lesions, accurate diagnosis can be difficult even for seasoned pathologists, let alone junior pathologists. Furthermore, the histopathologic grade is prone to subjectivity. Previous studies have showed interobserver variability in the staging and grading of BCa [6, 18, 19]. Therefore, an automated analysis system is in high demand in the pathological field, which could considerably alleviate the workload, improve the reproducibility and diagnostic accuracy.

In recent years, with the improvement of computation power and the growing availability of whole slide images (WSIs) [20, 21], artificial intelligence (AI) has attained significant achievements in a wide range of fields, especially in histopathological diagnosis [22, 23]. AI technology can mine subvisual image features from digital images that cannot be recognized by pathologists with the naked eye to enable disease diagnosis and prognosis prediction [24, 25]. Many AI-based diagnostic systems have been developed with encouraging results for clinical applications [26–31]. In particular, AI performed admirably in biopsy specimens, which are often fragmentary, scarce or without background tissues [32], indicating a promising application for low-quality specimens. To the best of our knowledge, few studies have applied deep learning to identify the pathological grading of NMIBC [33, 34]. The efficacy was not convincing enough due to the small sample size and low diagnostic accuracy, with limited clinical impact. Furthermore, there is no report on research regarding diagnosing muscle invasion, highlighting the necessity of this study.

Here, we report a pathological artificial intelligence diagnostic model (PAIDM) for BCa. The PAIDM not only showed excellent performance in diagnosing muscle invasion but also performed well in identifying histologic grade at the patch level and WSI level. We also compared the diagnostic accuracy between the PAIDM and pathologists.

## Methods

### Patients

In this study, a total of 716 consecutive patients with BCa from Sun Yat-sen Memorial Hospital (SYSMH) of Sun Yat-sen University were included. The patients underwent TURBT between January 2013 and November 2019 and were pathologically diagnosed with BCa, with detailed clinical and pathological data. The patients included were both newly diagnosed BCa patients and those who had priorly received Bacillus Calmette-Guerin or endovesical chemotherapy. The haematoxylin and eosin (H&E)-stained pathology slides of each patient were collected and scanned into WSIs at 40-fold magnification through an automatic digital slide scanner (KF-PRO-120/005, KFBIO Co., Ltd.). Low quality images due to extreme fading or low resolution, as well as those with both high-grade and low-grade tumour cells in the same image, were excluded. Finally, 854 pathological images of 692 patients passed quality control. This retrospective study was approved by the institutional review board of SYSMH, and the requirement for informed consent was waived. Clinicopathological characteristics were retrieved from the archives of medical records, and the details are shown in Table 1.

**Table 1:** Clinicopathologic characteristics of the patients in the training set and validation sets

	Training set (n = 313)	Validation set 1 (n = 95)	Validation set 2 (n = 201)	Validation set 3 (n = 83)
Age, Median (IQR), y	63 (56–72)	63 (55–72)	64 (57–71)	63 (56–70)
Sex				
Male	261 (83.4%)	78 (82.1%)	167 (83.1%)	70 (84.3%)
Female	52 (16.6%)	17 (17.9%)	34 (16.9%)	13 (15.7%)
T stage				
Ta	19 (6.1%)	3 (3.2%)	13 (6.5%)	10 (12.0%)
T1	207 (66.1%)	60 (63.2%)	148 (73.6%)	52 (62.7%)
T2-*	87 (27.8%)	32 (33.7%)	40 (19.9%)	21 (25.3%)
Grade				
Low grade	90 (28.8%)	28 (29.5%)	96 (47.8%)	35 (42.2%)
High grade	223 (71.2%)	67 (70.5%)	105 (52.2%)	48 (57.8%)

*IOR*  interquartile range. *T2- refer to at least T2

**Fig. 1:**
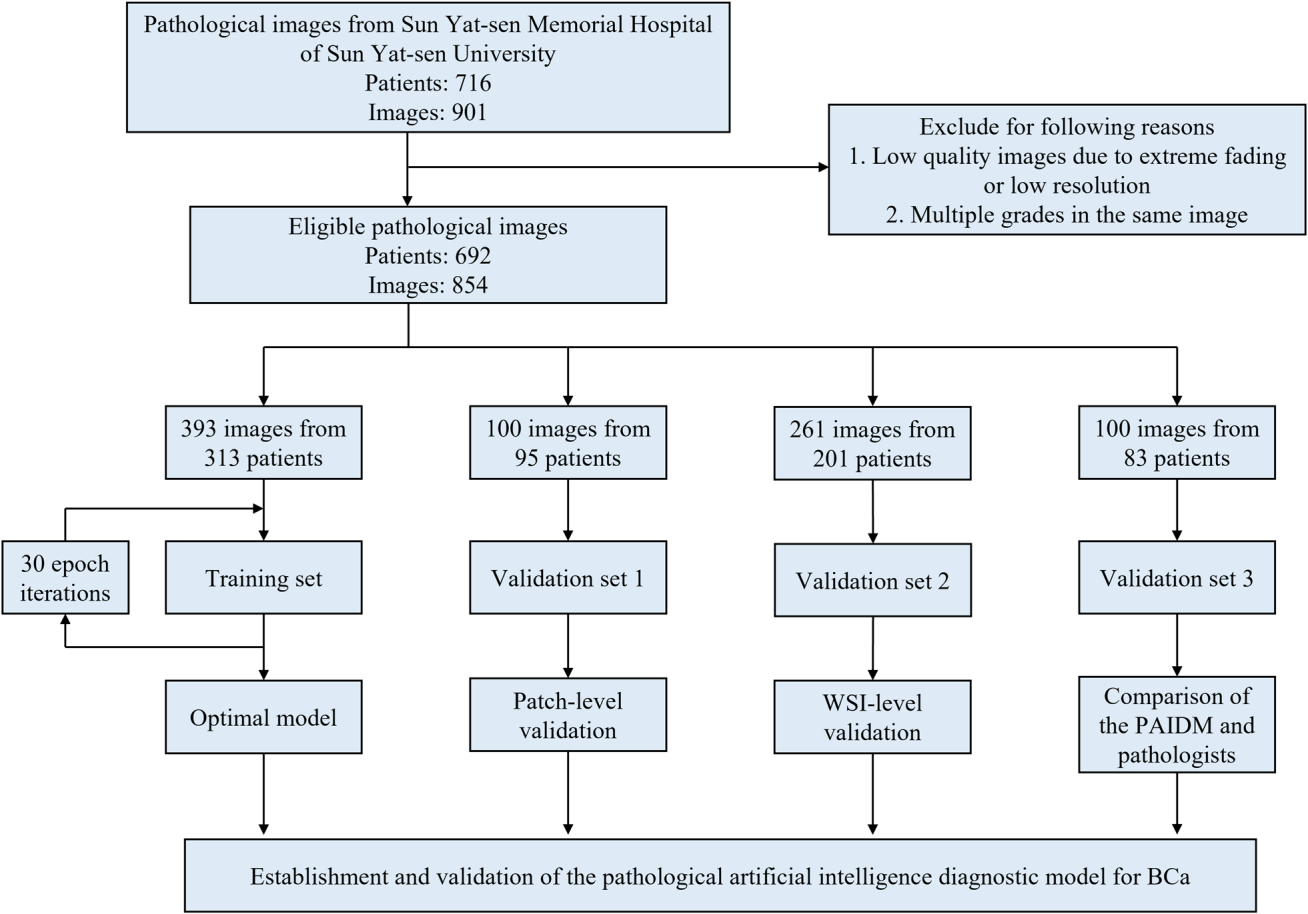
Study flowchart of the pathological artificial intelligence diagnostic model (PAIDM). *BCa*  bladder cancer, *WSI*  whole slide image

The images were randomly divided into two groups. One group was thoroughly annotated, whereas the other was not annotated and merely given category labels. We separated the group with full image annotation (493 images) into a training set and validation set 1 in a 4:1 ratio. The training set was used to develop the PAIDM, and the performance was evaluated at the patch level in validation set 1. The unannotated group (361 images) was separated into two validation sets: validation set 2 and validation set 3. Validation set 2 was used to evaluate the PAIDM’s performance at the WSI level, while validation set 3 was used for a comparison between the PAIDM and six pathologists. The study flowchart is shown in Fig. 1.

### Image annotation and preparation

All WSIs were obtained by the automatic scanner and stored in KFB format. To annotate the images conveniently, we uniformly converted all images into TIFF format. According to histologic grade, all MIBC is regarded as high-grade, while NMIBC comprises both low-grade and high-grade [35]. Therefore, we classified all images into three categories at the WSI level: high-grade muscle invasion (HGMI), high-grade non-muscle invasion (HGNMI) and low-grade non-muscle invasion (LGNMI). For fully annotated images, we also set six labels, including HGMI, HGNMI, LGNMI, illegible area (IA), normal interstitial area (NIA) and noise area (NA), at the patch level. The HGMI type had both high-grade tumour cells and bladder muscle tissue, whereas the HGNMI type only contained high-grade tumour cells and the LGNMI type only contained low-grade tumour cells. IA was defined as the blurred area of the image caused by scanning, NIA was defined as all normal mesenchymal cells, and NA was defined as the noncellular area owing to staining. The images were manually annotated by pathologists using automated slide analysis platform software (version 2.0), and the labels are presented in detail in Additional file 1: Fig. S1. Six experienced pathologists from SYSMH were divided into two groups for image classification and annotation, and a consensus reading by three pathologists in the same group was used. If they disagreed on the result of the image categories, the image was submitted to a pathologist with more than 30 years of expertise for reassessment.

WSIs are usually large in size and contain many white backgrounds without tissue. Previous research has found that on average, 82% of pathological images are backgrounds [36]. As a result, preprocessing is necessary to eliminate white backgrounds to increase the speed of diagnosis and analysis. The OTSU algorithm was used to determine the adaptive threshold for filtering out white backgrounds. First, we used a region of interest (ROI) with a size of 2048*2048 pixels and an adaptive threshold to binarize the image using the OTSU algorithm. The proportion of tissue area within the ROI was then calculated. If the proportion was less than the defined threshold, it was considered part of the white background and was not processed further. Additional file 1: Fig. S2a depicts the process of removing the white background. The red boxes are the preserved tissue area, and the white backgrounds were removed.

### Development of the PAIDM algorithm

The PAIDM was developed using the deep learning algorithm ScanNet, which has previously been used to efficiently identify and classify lymphatic metastasis [37]. For the categorization of the HGMI, HGNMI and LGNMI subtypes of BCa, we applied a convolutional neural network (CNN) based on ScanNet for rapid inference to match the speed requirements of clinical practice. The training set of 393 images was split into two parts: 318 images were used to train the PAIDM, and 75 images were used to fine-tune hyperparameters and optimize the model. During training procedure, the weights of ScanNet were initialized by ImageNet pre-trained model. Cross-entropy loss and stochastic gradient descent optimizer with a momentum of 0.9 and a weight decay of 0.0001 were selected to optimize the weights of network. The initial learning rate was 0.01 and multiplied by 0.1 on the 18th and 24th epochs. After 30 epochs of iteration, the PAIDM was developed. The learning curves of our model are shown in Additional file 1: Fig. S3.

In the training stage, six types of patches (HGMI, HGNMI, LGNMI, IA, NIA, and NA) were acquired from dataloader based on the txt file saved in the preprocessing stage (Additional file 1: Methods), and the patch-level classifier was trained. During the validation stage, the images were split into patches and then input into the CNN for classification. The outputs of the patches were spliced together to obtain heatmaps. After patch prediction, we reconstructed a 6-channel heatmap with the scale of 1/32 of original input size. Each point in heatmap was a patch prediction. We selected the channel with the largest probability as the decision class of this responding point. Then, the heatmap was used to generate contours for each single categorization and the area and mean probability of the contour were recorded. Eventually, we used the following formulas to determine the confidence of WSI-level categorization for each single class.

$${Prob}_{k}=\frac{{e}^{{P}_{k}}}{\sum_{k}^{n}{e}^{{P}_{k}}}$$

where $k$ represents the specific class, $n$ represents the number of categorizations, ${P}_{k}$ is an intermediate value, followed as:

$${P}_{k}=\frac{\sum_{i=1}^{m}{a}_{i}*{p}_{i}}{\sum_{i=1}^{m}{a}_{i}}$$

where $m$ represents the number of contours, ${a}_{i}$ is the i-th contour area for a single class, ${p}_{i}$ is the mean probability of all points in the i-th contour area.

**Fig. 2:**
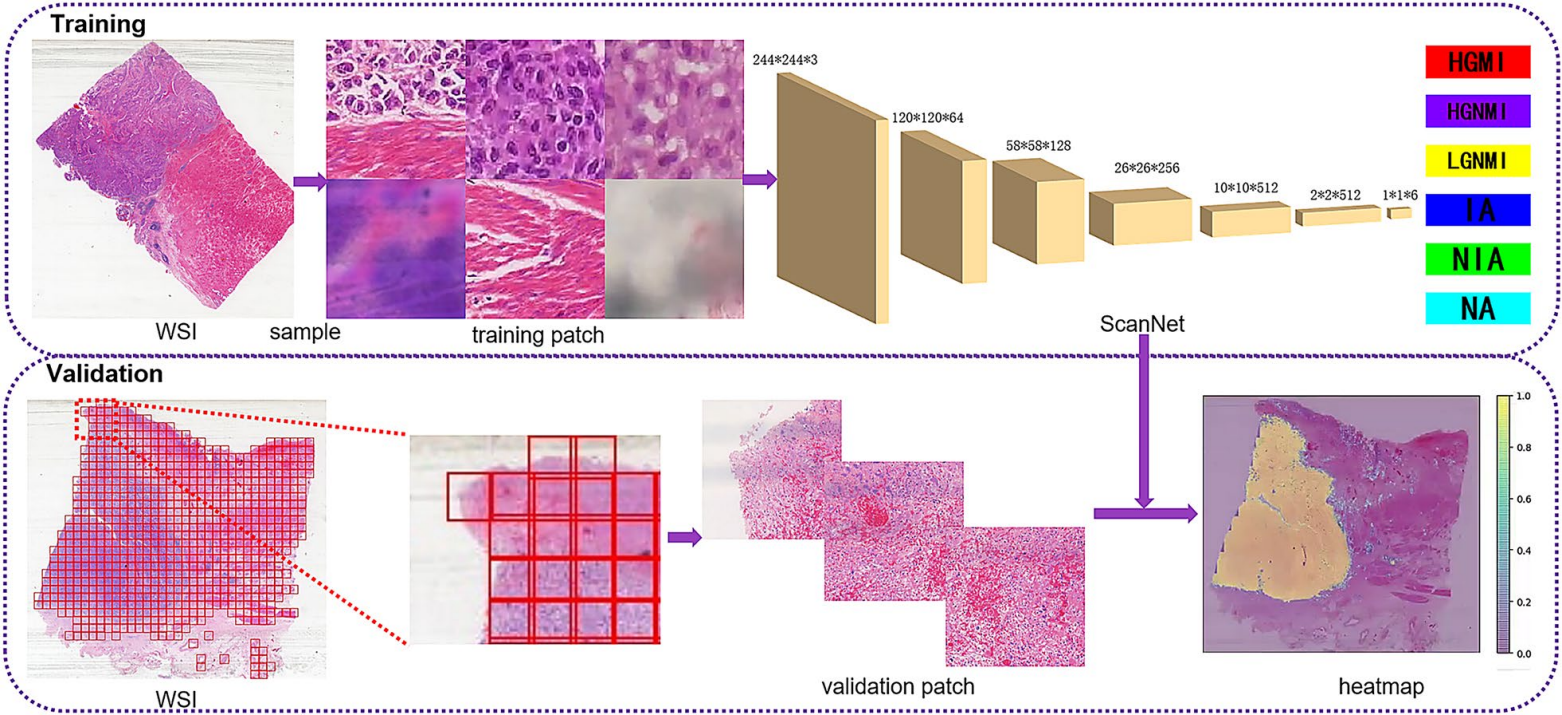
Diagram for the development and validation of the pathological artificial intelligence diagnostic model. In the training stage, a CNN model (ScanNet) was trained with the training patch, and the patch-level classifier was developed. In the validation stage, the WSI was first divided into validation patches and then input into ScanNet. The outputs of the patches were spliced together to obtain heatmaps. The probability weighted value of each subtype was calculated to give the confidence of WSI-level classification. *CNN*  convolutional neural network, *WSI*  whole slide image, *HGMI*  high-grade muscle invasion, *HGNMI*  high-grade non-muscle invasion, *LGNMI*  low-grade non-muscle invasion, *IA*  illegible area, *NIA*  normal interstitial area, *NA*  noise area

The diagram is shown in Fig. 2, and the algorithm is described in detail in the Additional file 1. To train and evaluate the PAIDM, we adopted an Ubuntu 16.04 computer and used PyTorch within the Python (version 3.8) programming language. The hardware component we used was a GeForce GTX TITAN X GPU with 12-GB memory.

### Performance validation of the PAIDM

**Fig. 3:**
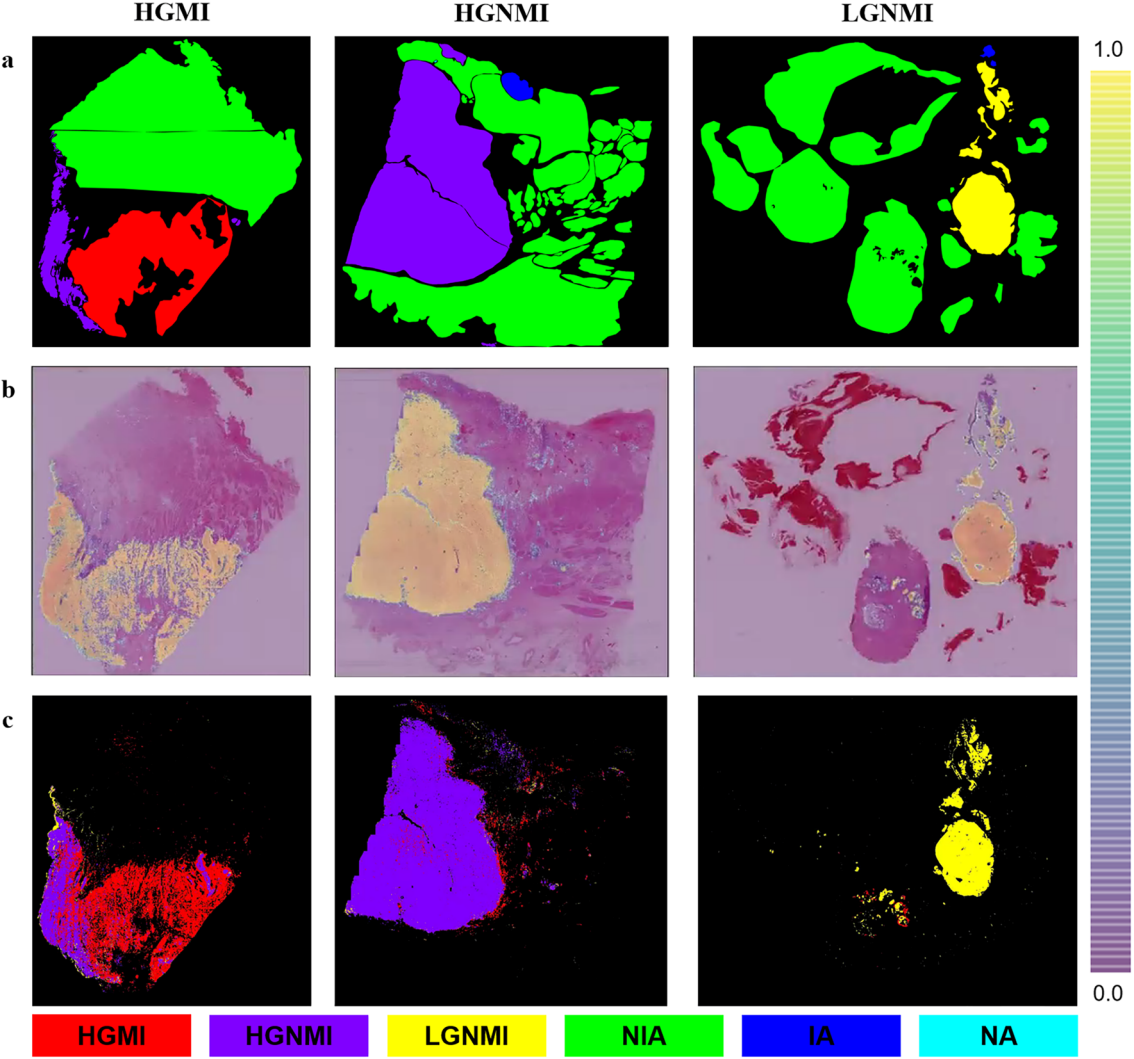
Examples of manual annotation and automatic tissue segmentation. **a** Manual annotation by pathologists. **b** Heatmaps generated by the PAIDM. **c** Masks predicted by the PAIDM. *PAIDM*  pathological artificial intelligence diagnostic model, *HGMI*  high-grade muscle invasion, *HGNMI*  high-grade non-muscle invasion, *LGNMI*  low-grade non-muscle invasion, *IA* illegible area, *NIA*  normal interstitial area, *NA*  noise area

To assess the PAIDM’s performance, the confusion matrix and receiver operating characteristic (ROC) curves were used. The area under the curve (AUC), accuracy, sensitivity, specificity, positive predictive value (PPV) and negative predictive value (NPV), as well as their 95% confidence intervals (CIs), were calculated using the Clopper-Pearson method. Furthermore, to better understand the PAIDM categorization, we used heatmaps to visualize the predictions of the HGMI, HGNMI and LGNMI subtypes to verify whether the information used for categorization was reasonable. The heatmaps are shown in Fig. 3.

We evaluated the classification performance at the patch level in validation set 1 and at the WSI level in validation set 2. For the comparison between the PAIDM and pathologists, an independent validation cohort (validation set 3) was employed. Six pathologists with varying levels of clinical expertise (two junior pathologists with approximately 5 years of experience, two intermediate pathologists with more than 10 years of experience, and two senior pathologists with at least 15 years of experience) were asked to independently diagnose every image. The diagnostic results of the tested images were not disclosed to any of the six pathologists, and none of them was involved in other parts of this study.

### Statistical analysis

The statistical analysis was implemented based on Python (version 3.8). The open-source scikit-learn toolkit was used to analyse the AUC, accuracy, sensitivity, specificity, PPV and NPV. All statistical tests were two-sided, and *P* < 0.05 was considered statistically significant. The CIs were at the 95% level.

## Results

### Clinicopathological characteristics of the included patients

In total, 854 images from 692 patients were included to develop and validate the PAIDM. Specifically, the training set was used to train and optimize the PAIDM. The PAIDM’s performance was evaluated at the patch level in validation set 1 and at the WSI level in validation set 2. In addition, an independent validation cohort (100 images) was used for the comparison between the PAIDM and pathologists. Additional file 1: Fig. S4 shows the proportion of patients with HGMI, HGNMI and LGNMI subtypes in training set and three validation sets.

Clinicopathological characteristics, including age, sex, T stage and histologic grade, are shown in Table 1. In all four datasets, there were considerably more male patients than female patients, with a male-to-female ratio close to 4:1. This was consistent with the fact that BCa is more common in males. NMIBC contains stages Ta and T1, whereas MIBC refers to T2-. The proportion of NMIBC was approximately 70%, while that of MIBC was approximately 30%, which was roughly comparable to the data reported previously [38]. Moreover, the proportion of patients with high-grade BCa was larger than that of patients with low-grade BCa.

### Patch-level classification performance in validation set 1

**Fig. 4:**
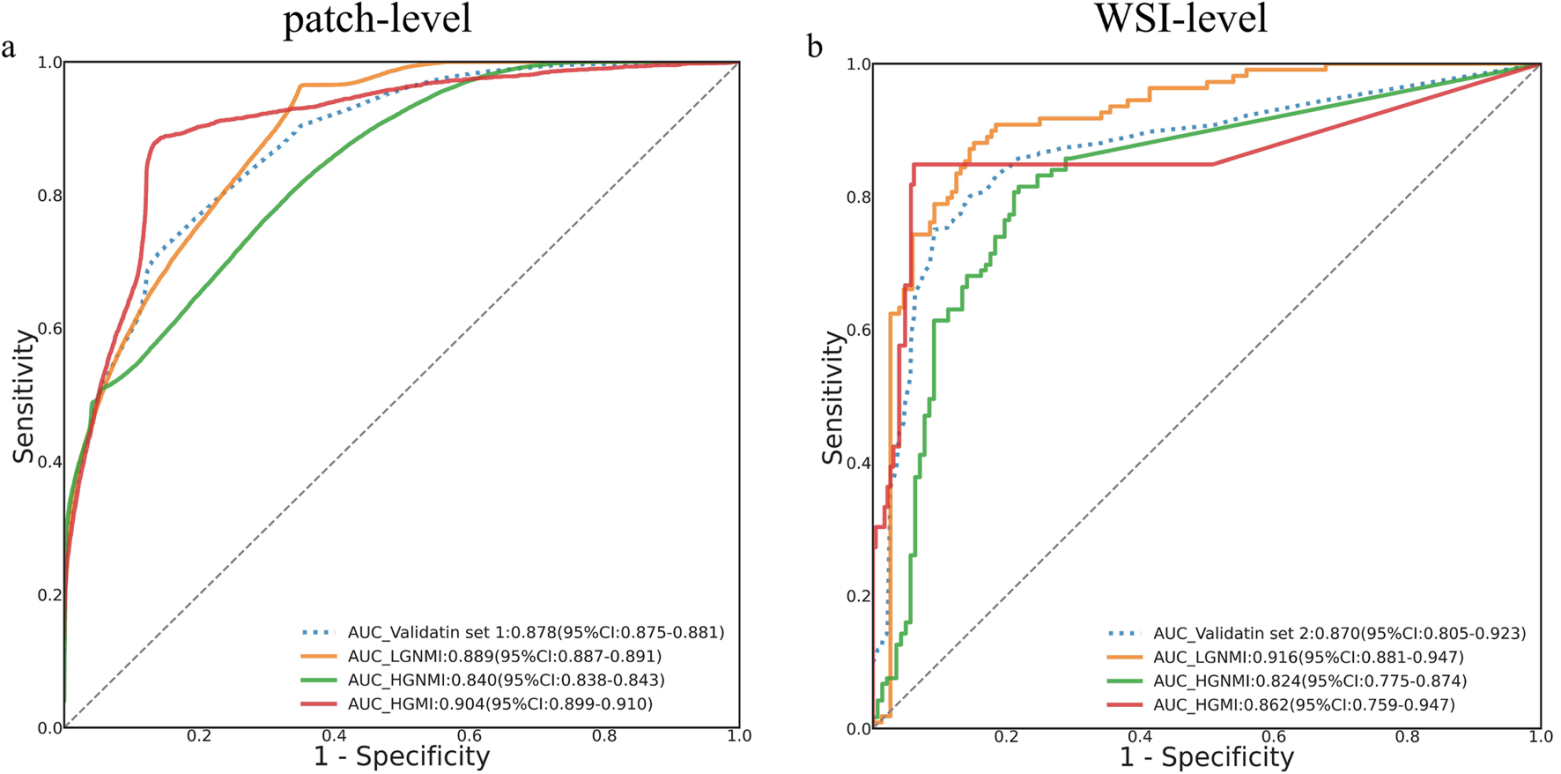
Performance of the PAIDM in two validation sets. **a** ROC curves for patch-level diagnostic performance in validation set 1. **b** ROC curves for WSI-level classification performance in validation set 2. *PAIDM*  pathological artificial intelligence diagnostic model, *ROC*  receiver operating characteristic, *WSI*  whole slide image, *AUC*  area under the curve, *HGMI*  high-grade muscle invasion, *HGNMI*  high-grade non-muscle invasion, *LGNMI*  low-grade non-muscle invasion

Due to the poor quality of transurethral resection specimens, which are frequently fragmented, scarce or lacking a full muscle layer, even experienced pathologists might make a misdiagnosis. To evaluate the PAIDM’s diagnostic performance for limited tissues, a total of 112,472 patches from 100 WSIs were collected to test the patch-level recognition capability. The diagnostic criteria were defined based on the preceding annotation labels. As shown in Fig. 4a, the PAIDM achieved a favourable recognition capability, with a patch-level AUC of 0.878 (95% CI 0.875–0.881) in the classification task. The AUCs of the LGNMI, HGNMI and HGMI subtypes were 0.889 (95% CI 0.887–0.891), 0.840 (95% CI 0.838–0.843), and 0.904 (95% CI 0.899–0.910), respectively, indicating a significant clinical application for sparse specimens. In addition, Additional file 1: Fig. S5 shows the PAIDM’s performance for WSI-level diagnosis in validation set 1.

### WSI-level diagnostic performance in validation set 2

To evaluate the PAIDM’s performance at the WSI level, another batch of 261 WSIs was used for the multi-classification task. As shown in Fig. 4b, the PAIDM achieved an overall AUC of 0.870 (95% CI 0.805–0.923) for the three categories overall. The PAIDM performed well in identifying LGNMI and HGMI with AUCs of 0.916 (95% CI 0.881–0.947) and 0.862 (95% CI 0.759–0.947), respectively, while it performed slightly worse in detecting HGNMI, with an AUC of 0.824 (95% CI 0.775–0.874). Regarding the diagnosis of MIBC, the PAIDM maintained a satisfactory identification performance, with an accuracy of 0.850 (95% CI 0.777–0.924), a specificity of 0.941 (95% CI 0.912–0.969) and an NPV of 0.963 (95% CI 0.943–0.982).

Furthermore, in the grading task of low-grade and high-grade BCa, the PAIDM also showed excellent capability, with an accuracy of 0.862 (95% CI 0.818–0.905). The sensitivity and specificity were 0.867 (95% CI 0.807–0.927) and 0.849 (95% CI 0.790–0.908), respectively. Additional file 1: Table S1 presents the indexes, including the accuracy, sensitivity, specificity, PPV and NPV, which were calculated from the confusion matrix (Additional file 1: Fig. S6).

### WSI-level heatmaps of classification prediction

To better comprehend the PAIDM categorization, we visualized the predictive results using heatmaps, which showed the classification prediction of the HGMI, HGNMI and LGNMI subtypes. Figure 3 illustrates examples of manual annotation and automatic tissue segmentation. Figure 3a shows the masks annotated manually by pathologists, while Fig. 3b-c shows the heatmaps and masks generated by the CNN model. The red area indicates the tissue of HGMI, whereas the purple area represents HGNMI, and the yellow area represents LGNMI. As seen in Fig. 3, there was high consistency between the model-predicted and manually annotated areas for each category, indicating that the information utilized by the PAIDM for categorization is reasonable. Furthermore, by highlighting prediction masks in the image, the PAIDM can assist pathologists in focusing on suspicious regions faster and improving diagnostic efficiency.

### Comparison with pathologists in validation set 3

**Fig. 5:**
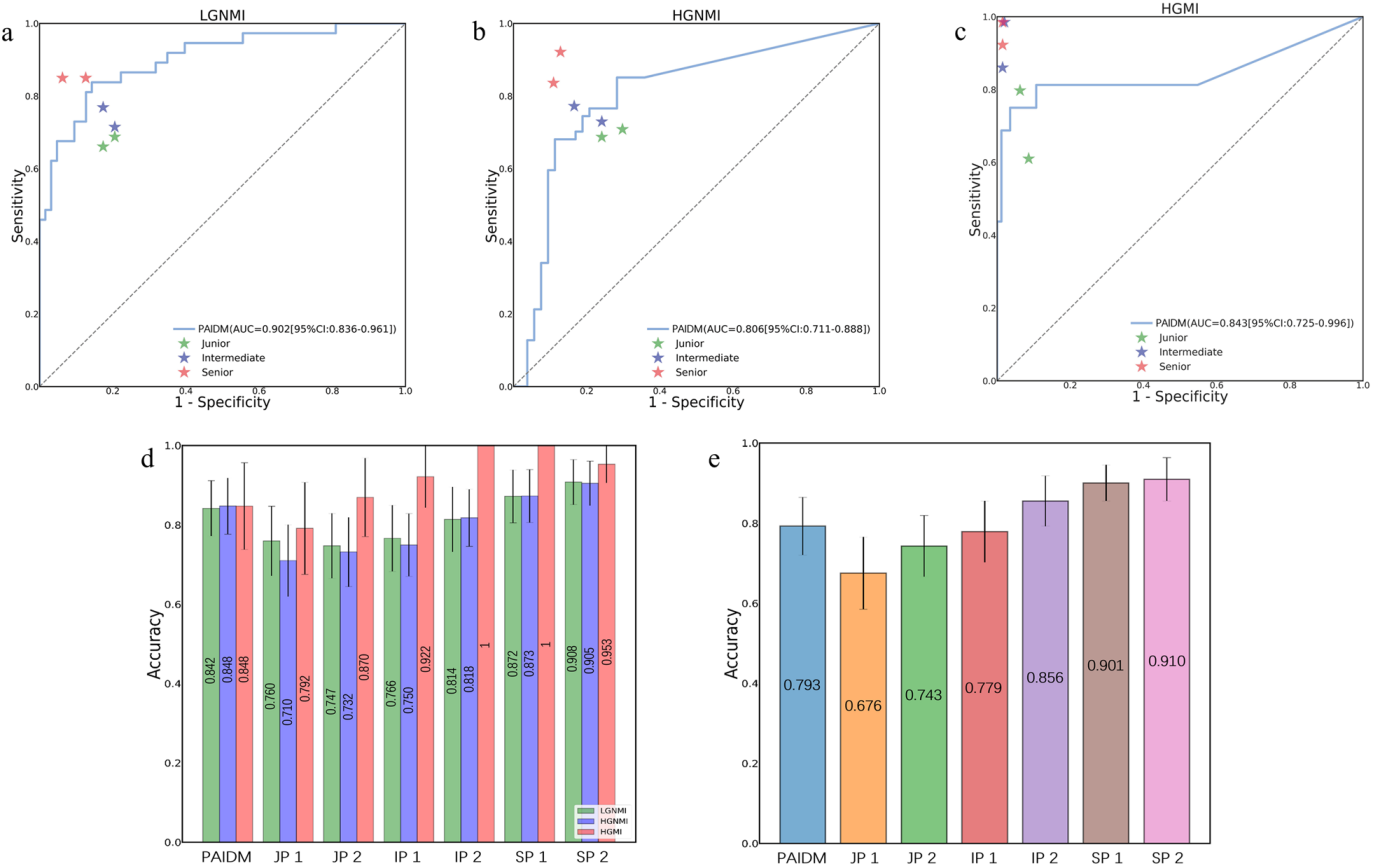
Comparison between the PAIDM and pathologists. **a** ROC curve for the performance of the PAIDM versus six pathologists in identifying LGNMI. **b** ROC curve for the performance of the PAIDM versus six pathologists in identifying HGNMI. **c** ROC curve for the performance of the PAIDM versus six pathologists in identifying HGMI. **d** Diagnostic accuracy of the PAIDM versus six pathologists in classifying the LGNMI, HGNMI and HGMI subtypes. **e** The average accuracy of the PAIDM versus six pathologists in the classification task. Error bars represent the 95% confidence intervals. *PAIDM*  pathological artificial intelligence diagnostic model, *ROC*  receiver operating characteristic, *AUC*  area under the curve, *LGNMI*  low-grade non-muscle invasion, *HGNMI*  high-grade non-muscle invasion, *HGMI*  high-grade muscle invasion, *JP*  junior pathologist, *IP*  intermediate pathologist, *SP*  senior pathologist

For validation purposes, an independent validation cohort (validation set 3) was used to evaluate the diagnostic performance of the PAIDM and pathologists. As shown in Table 2, the AUCs of the two junior, two intermediate and two senior pathologists were 0.752 (95% CI 0.644–0.846), 0.792 (95% CI 0.697–0.877), 0.822 (95% CI 0.741–0.897), 0.877 (95% CI 0.826–0.935), 0.918 (95% CI 0.876–0.957) and 0.930 (0.865–0.976), respectively. By contrast, the PAIDM achieved an AUC of 0.847 (95% CI 0.779–0.905), which performed better than the junior pathologists, comparable to the intermediate pathologists, and slightly worse than the senior pathologists. On average, it took the junior, intermediate, and senior pathologists 252 s, 195 s, and 178 s, respectively, to review each WSI, whereas the average inference time of the PAIDM was 144 s per image, which was shortened compared to pathologists at all levels. Figure 5a-c shows the performance of PAIDM versus six pathologists in identifying the LGNMI, HGNMI and HGMI subtypes, while the histogram in Fig. 5d-e shows the accuracy comparison between the PAIDM and pathologists. Additional file 1: Table S2 presents the diagnostic accuracy of the PAIDM and pathologists in validation set 3.

**Table 2:** Comparison between the PAIDM and pathologists

	AUC (95% CI)	Accuracy (95% CI)	Sensitivity (95% CI)	Specificity (95% CI)	PPV (95% CI)	NPV (95% CI)
PAIDM	0.847 (0.779–0.905)	0.793 (0.721–0.865)	0.797 (0.721–0.874)	0.899 (0.860–0.937)	0.816 (0.759–0.872)	0.902 (0.869–0.935)
Junior pathologist 1	0.752 (0.644–0.846)	0.676 (0.586–0.766)	0.680 (0.595–0.766)	0.838 (0.788–0.887)	0.709 (0.634–0.783)	0.842 (0.803–0.882)
Junior pathologist 2	0.792 (0.697–0.877)	0.743 (0.667–0.820)	0.734 (0.658–0.811)	0.865 (0.820–0.910)	0.747 (0.680–0.813)	0.869 (0.835–0.903)
Intermediate pathologist 1	0.822 (0.741–0.897)	0.779 (0.703–0.856)	0.779 (0.703–0.856)	0.890 (0.851–0.928)	0.803 (0.749–0.856)	0.891 (0.858–0.924)
Intermediate pathologist 2	0.877 (0.826–0.935)	0.856 (0.793–0.919)	0.860 (0.802–0.919)	0.928 (0.901–0.955)	0.862 (0.815–0.908)	0.931 (0.905–0.958)
Senior pathologist 1	0.918 (0.876–0.957)	0.901 (0.856–0.946)	0.901 (0.847–0.955)	0.951 (0.923–0.978)	0.905 (0.860–0.951)	0.953 (0.928–0.977)
Senior pathologist 2	0.930 (0.865–0.976)	0.910 (0.856–0.964)	0.910 (0.856–0.964)	0.955 (0.928–0.982)	0.920 (0.876–0.964)	0.957 (0.931–0.982)

*PAIDM*  pathological artificial intelligence diagnostic model, *AUC*  area under the curve, *PPV*  positive predictive value, *NPV*  negative predictive value, *CI*  confidence interval

## Discussion

In this study, we initially reported an AI-based pathological diagnostic model for transurethral resection specimens of BCa, designated PAIDM. The PAIDM not only showed excellent performance in identifying MIBC but also performed well in distinguishing high-grade and low-grade BCa. More importantly, the PAIDM excelled at both WSI-level and patch-level recognition, with a promising application for BCa staging and grading.

The accurate diagnosis of invasion depth and histologic grade is critical for clinical management in BCa patients. However, some dilemmas still remain regarding the pathological diagnosis, such as the misdiagnosis of MIBC and interobserver variability. Erroneous staging of BCa can result in an omission or delay in providing optimal treatment, leading to disease progression and tumour recurrence [39]. For example, if a patient with MIBC is misdiagnosed as NMIBC, optimal treatment, such as radical cystectomy and neoadjuvant chemotherapy, cannot be implemented in time, which is likely to lead to a poor clinical outcome. In our study, the PAIDM achieved satisfactory performance in identifying muscle invasion at the WSI level, with an accuracy of 0.850 and a specificity of 0.941. The PAIDM had a relatively low sensitivity of 0.743. The underlying reason might be that some specimens lacked a complete muscle layer and the model failed to extract effective features, leading to missed diagnoses of MIBC. It is worth noting that the PAIDM also performed well at patch-level recognition, with an AUC of 0.904, indicating a significant clinical application for some specimens with minimal tissue.

With the rise in cancer morbidity, there is a significant shortage of pathologists to meet the growing demands for diagnosis. In clinical practice, pathologists have to deal with many cases and review associated pathology slides to confirm cancer diagnosis every day, which is labour-intensive and time-consuming. For atypical or complex cases, pathologists are prone to subjectivity with significant inter- and intra-observer variability, which greatly relies on the skills and experiences of pathologists. As a result, there is a high demand for automated analytic systems to reduce the burden, increase diagnostic consistency and reliability. In clinical practice, our system enables a completely automated and integrated diagnosis process. The pathologists only need to put a batch of stained slides into the scanner, and the scanner will automatically complete the scanning, upload the WSIs to the diagnostic platform and realize the end-to-end diagnostic output, without additional manual involvement. It could handle vast amounts of images effectively and is less prone to fatigue, with better reproducibility and stability. Furthermore, our system could not only provide diagnosis outcomes, but also highlight prediction masks in the images, allowing pathologists to visualize the inference results and aid in focusing on suspicious regions to improve diagnostic efficiency.

Additionally, the PAIDM is a practical tool for bridging the diagnostic gap between national hospitals and primary care hospitals, as well as the gap between experienced pathologists and junior pathologists. In the comparison between the PAIDM and pathologists, the PAIDM was non-inferior to the average diagnostic level of pathologists, reaching the intermediate expert level, indicating that the PAIDM might improve the diagnostic accuracy of inexperienced pathologists, particularly junior pathologists. Although the PAIDM did not reach the level of senior experts, all the intermediate pathologists in the comparison came from the first-rate hospital in China, and all of them had more than 10 years of clinical expertise, so we assume that they are no less competent than the experts in municipal or grass-roots hospitals. Hence, in China, where medical resources are unbalanced between urban and rural areas, we can apply the PAIDM in developed areas to improve the diagnostic efficiency of experienced experts and in remote areas to improve the diagnostic accuracy of inexperienced pathologists, thereby promoting medical care homogenization. It is worth mentioning that we believe that AI-based diagnostic models are currently used as an adjunct rather than a replacement.

According to reports, few previous studies have applied deep learning for the pathological grading of NMIBC. Ilaria Jansen et al. developed an automated detection and grading model for classifying low-grade and high-grade BCa [33]. Peng-Nien Yin et al. used six machine learning approaches to distinguish stage Ta and stage T1 BCa [34]. However, the efficacy of the above models was not convincing enough due to the small sample size and low diagnostic accuracy, which limited their clinical application. Compared with the previous models, our model was based on a larger dataset and achieved a higher accuracy for the pathological grading of BCa. Additionally, the images included for training were completely annotated, thus making full use of the information in each pathological image. More notably, the PAIDM performed well in identifying MIBC, which had not been achieved in previous studies but is vital for clinical decision making. To our knowledge, this is the first study to apply AI for the pathological diagnosis of muscle invasion in BCa.

Although our model achieved remarkable results, some limitations still must be addressed. First, since this study was single-centre and retrospective, the issue of overfitting needs to be thoroughly considered. Despite the fact that we applied data enhancement strategies such as translation, rotation, scaling, flipping and colour jitter to improve the robustness, multi-centre and prospective studies are still needed for further validation. The generalizability of the PAIDM can be boosted by increasing the amount and diversity of the samples. Second, the annotation method used in this study was full annotation, which fully utilized the information of each pathological image; however, the labelling work was time-consuming and it was difficult to include more images for training. It will be critical to incorporate annotated data based on partial annotation and weak supervision [40] to further improve the performance. Third, although carcinoma in situ is NMIBC, it is poorly differentiated and has a high risk of disease progression. The PAIDM is not yet able to classify this type effectively, and further optimization of the model with the inclusion of this type of data is needed. It is worth noting that our original intention was to design a model to diagnose histologic grade and muscle invasion or not, so we paid more attention to bladder cancer itself. However, in clinical work, the diagnoses of other lesions such as dysplasia and inflammation are also important. We plan to add such samples to further train the model in the future, so as to improve the applicability of the model.

## Conclusions

In conclusion, we developed an PAIDM for the pathological diagnosis of BCa, with an encouraging result. More significantly, the PAIDM performed admirably at patch-level recognition, which may be helpful for fragmented specimens. It is expected to be applied as a reliable pathology-assisted diagnostic tool in clinic.

## Supplementary Information

**Additional file 1: Table S1.** WSI-level diagnostic performance of the PAIDM in validation set 2. **Table 2.** Diagnostic accuracy of the PAIDM and pathologists in validation set 3. **Figure S1.** Examples of six labels for fully annotated images. (a) HGMI contained both high-grade tumour cells and bladder muscle tissue. (b) HGNMI only contained high-grade tumour cells. (c) LGNMI only contained low-grade tumour cells. (d) IA was defined as the blurred area due to scanning. (e) NIA included all normal mesenchymal cells. (f) NA indicated the noncellular area due to staining. HGMI=high-grade muscle invasion. HGNMI=high-grade non-muscle invasion. LGNMI=low-grade non-muscle invasion. IA=illegible area. NIA=normal interstitial area. NA=noise area. **Figure S2.** Image preprocessing and patch sampling. (a) The OTSU algorithm was used to eliminate the white backgrounds to improve the efficiency of diagnosis and analysis. The red boxes are the preserved tissue area, and the white backgrounds were removed. (b) The sliding window method was used to extract patches of the HGNMI, LGNMI, IA, NIA and NA types. The sampling points were within the annotation region to guarantee that each patch comprised just the certain kind of tissue. (c) The point-based labelling method was adopted to extract the typical patches of HGMI. The sampling points were marked at the junction of tumour tissue and muscle tissue. The green points are the sampling points of the HGMI type. HGMI=high-grade muscle invasion. HGNMI=high-grade non-muscle invasion. LGNMI=low-grade non-muscle invasion. IA=illegible area. NIA=normal interstitial area. NA=noise area. **Figure S3. **Learning curves of the PAIDM. The learning curves of the PAIDM show the gradual decrease of loss (a) and increase of accuracy (b) during the training process. PAIDM=pathological artificial intelligence diagnostic model. **Figure S4**. The proportion of patients with HGMI, HGNMI and LGNMI subtypes in training set and three validation sets. HG= high grade. LG= low grade. NMIBC=non-muscle-invasive bladder cancer. MIBC= muscle-invasive bladder cancer. **Figure S5.** ROC curves for WSI-level diagnostic performance of the PAIDM in validation set 1. PAIDM=pathological artificial intelligence diagnostic model. WSI= whole slide image. ROC=receiver operating characteristic. AUC=area under the curve. LGNMI=low-grade non-muscle invasion. HGNMI=high-grade non-muscle invasion. HGMI=high-grade muscle invasion. **Figure S6.** Confusion matrices of the PAIDM in the two validation sets. (a) Confusion matrix for the patch-level classification in validation set 1. (b) Confusion matrix for the WSI-level classification in validation set 1. (c) Confusion matrix for the WSI-level classification in validation set 2. PAIDM=pathological artificial intelligence diagnostic model. WSI= whole slide image. LGNMI=low-grade non-muscle invasion. HGNMI=high-grade non-muscle invasion. HGMI=high-grade muscle invasion. **Figure S7.** Diagnostic parameters to assess the PAIDM at the WSI level in validation set 2. AUC=area under the curve. CI=confidence interval. PPV=positive predictive value. NPV=negative predictive value. HG= high grade. LG= low grade. NMIBC=non-muscle-invasive bladder cancer. MIBC= muscle-invasive bladder cancer. PAIDM=pathological artificial intelligence diagnostic model. WSI=whole slide image. **Figure S8.** Diagnostic performance of the PAIDM in six-class recognition at the patch level in validation set 1. (a) ROC curves for patch-level diagnostic performance of six classes. (b) Confusion matrix for patch-level diagnostic performance of six classes. PAIDM=pathological artificial intelligence diagnostic model. ROC=receiver operating characteristic. AUC=area under the curve. HGMI=high-grade muscle invasion. HGNMI=high-grade non-muscle invasion. LGNMI=low-grade non-muscle invasion. IA=illegible area. NIA=normal interstitial area. NA=noise area.

## Data Availability

The source codes used in this study are available online. To protect the privacy of the patients, the pathological image dataset and other data related to patients cannot be made available for public access, but all data are available from the corresponding author upon reasonable request. Data sharing requests can be sent to lintx@mail.sysu.edu.cn by email. To gain access, data requestors will need to sign a data access agreement.
