# Supplementary material for: An artificial intelligence model for the pathological diagnosis of invasion depth and histologic grade in bladder cancer
Source: J Transl Med. 2023 Jan 23;21:42. doi: 10.1186/s12967-023-03888-z (PMC9869632; doi:10.1186/s12967-023-03888-z)
Supplement: Supplementary file 1 — Additional file 1: Table S1. WSI-level diagnostic performance of the PAIDM in validation set 2. Table 2. Diagnostic accuracy of the PAIDM and pathologists in validation set 3. Figure S1. Examples of six labels for fully annotated images. (a) HGMI contained both high-grade tumour cells and bladder muscle tissue. (b) HGNMI only contained high-grade tumour cells. (c) LGNMI only contained low-grade tumour cells. (d) IA was defined as the blurred area due to scanning. (e) NIA included all normal mesenchymal cells. (f) NA indicated the noncellular area due to staining. HGMI=high-grade muscle invasion. HGNMI=high-grade non-muscle invasion. LGNMI=low-grade non-muscle invasion. IA=illegible area. NIA=normal interstitial area. NA=noise area. Figure S2. Image preprocessing and patch sampling. (a) The OTSU algorithm was used to eliminate the white backgrounds to improve the efficiency of diagnosis and analysis. The red boxes are the preserved tissue area, and the white backgrounds were removed. (b) The sliding window method was used to extract patches of the HGNMI, LGNMI, IA, NIA and NA types. The sampling points were within the annotation region to guarantee that each patch comprised just the certain kind of tissue. (c) The point-based labelling method was adopted to extract the typical patches of HGMI. The sampling points were marked at the junction of tumour tissue and muscle tissue. The green points are the sampling points of the HGMI type. HGMI=high-grade muscle invasion. HGNMI=high-grade non-muscle invasion. LGNMI=low-grade non-muscle invasion. IA=illegible area. NIA=normal interstitial area. NA=noise area. Figure S3. Learning curves of the PAIDM. The learning curves of the PAIDM show the gradual decrease of loss (a) and increase of accuracy (b) during the training process. PAIDM=pathological artificial intelligence diagnostic model. Figure S4. The proportion of patients with HGMI, HGNMI and LGNMI subtypes in training set and three validation sets. HG= high grade. LG= low [file 12967_2023_3888_MOESM1_ESM.docx]

**Additional Materials**

**An Artificial Intelligence Model for the Pathological Diagnosis of Invasion Depth and Histologic Grade in Bladder Cancer**

Jiexin Pan, MD^a,b,1^, Guibin Hong, MD^a,b,1^, Hong Zeng, MD^c,1^, Chengxiao Liao, MD^a^, Huarun Li, MD^a^, Yuhui Yao, MD^a^, Qinghua Gan, MD^a^, Yun Wang, MD^a^, Shaoxu Wu, PhD^a,b,d,*^, Tianxin Lin, PhD^a,b,d,*^

**Additional methods**

**Image patch sampling**

A digital pathological image is approximately 50000*50000 pixels in size. If the image is directly input into the CNN to extract features, the requirement of computation and memory space will far exceed the capability of the current mainstream GPU. Many details of cell morphology and organization characteristics will be lost if the image is downsampled to the point where the CNN can be carried out. As a result, we adopted the method of patch sampling^1^, which divided the image into multiple small-size patches. The patches were then input into the CNN to extract features, which were aggregated to generate the WSI features.

It is worth mentioning that we employed a region-based annotation approach, and the model was trained based on patches. To extract the corresponding types of patches, we needed to determine the sampling points within the annotation region. To guarantee that each patch comprised just the certain kind of tissue, we used the sliding window method to extract patches of the HGNMI, LGNMI, IA, NIA, and NA types. The sampling points are shown in Fig. S2b.

For the HGMI type, the patch extraction method described above did not achieve satisfactory results. The reason was that the HGMI type comprised both high-grade tumour cells and muscle tissue, but the sliding window method may extract patches of all the high-grade tumour cells or all the muscle tissue, which was classified as HGNMI or NIA. Therefore, we adopted the point-based labelling method to mark dots at the junction of tumour tissue and muscle tissue and then extracted the typical patches of HGMI with the sampling points as the centre. The green points in Fig. S2c are the sampling points of the HGMI type.

**Image preprocessing**

Due to the limited computing resources and the large size of pathological images, a lot of computation is required when using the OTSU algorithm to determine the threshold for directly excluding the white background on an ROI of 2048*2048 pixels. To speed up this process, we performed this operation on the downsampled image. We downsampled pathological images 16 times first and then performed the above operation. The corresponding ROI size was 128*128 pixels (2048/16), and the patch size was 16*16 pixels (256/16), thus greatly reducing the computational requirements and speeding up the preprocessing.

In each reserved ROI, patches of size 256*256 pixels with no overlapping region among them were selected. If there was tissue within the patches, these patches were reserved as the training patches, whereas those that did not contain tissue were excluded, further filtering out the white background. The label of the reserved patch was determined by the label marked by pathologists.

We stored the central coordinate information of each patch and its label in the txt file so that different types of training patches could be extracted based on the information saved in the preprocessing stage instead of prestoring the cut patches, thus reducing the memory space needed.

**Algorithm training strategies**

During the development of the PAIDM, we used strategies including asynchronous sample prefetching, data enhancement and hard negative mining strategies. In the training phase, we adopted the asynchronous sampling prefetch, which meant that multiple threads of CPU processed training samples at the same time to generate multiple patches, thus making full use of computing resources and improving the training efficiency. In addition, data enhancement^2^ played an important role in alleviating the overfitting problem. To improve the classification performance of the PAIDM, we processed the training patches using data augmentation methods such as translation, rotation, scaling, flipping and colour jitter. The hard negative mining strategy was employed in the false positive areas to re-add the hard samples of false positives to the training set and improve the PAIDM's performance by repeatedly identifying these hard sample data.

**Additional references**

1 Cruz-Roa A, Basavanhally A, Gonzalez F, Gilmore H, Feldman M, Ganesan S *et al.* Automatic detection of invasive ductal carcinoma in whole slide images with Convolutional Neural Networks. In: Gurcan M, Madabhushi A (eds). . 2014 doi:10.1117/12.2043872.

2 Tellez D, Litjens G, Bándi P, Bulten W, Bokhorst J-M, Ciompi F *et al.* Quantifying the effects of data augmentation and stain color normalization in convolutional neural networks for computational pathology. *Medical Image Analysis* 2019; **58**: 101544.

**Table S1.** WSI-level diagnostic performance of the PAIDM in validation set 2.

|  | Accuracy  (95% CI) | Sensitivity  (95% CI) | Specificity  (95% CI) | PPV  (95% CI) | NPV  (95% CI) |
| --- | --- | --- | --- | --- | --- |
| PAIDM_AVERAGE | 0.798  (0.755-0.841) | 0.797  (0.755-0.838) | 0.898  (0.876-0.919) | 0.803  (0.768-0.837) | 0.900  (0.881-0.918) |
| LGNMI | 0.862  (0.818-0.905) | 0.867  (0.807-0.927) | 0.849  (0.790-0.908) | 0.814  (0.754-0.874) | 0.902  (0.858-0.946) |
| HGNMI | 0.829  (0.785-0.873) | 0.761  (0.689-0.832) | 0.894  (0.844-0.943) | 0.862  (0.800-0.923) | 0.818  (0.771-0.866) |
| HGMI | 0.850  (0.777-0.924) | 0.743  (0.606-0.879) | 0.941  (0.912-0.969) | 0.671  (0.548-0.794) | 0.963  (0.943-0.982) |

WSI=whole slide image. PAIDM=pathological artificial intelligence diagnostic model. LGNMI=low-grade non-muscle invasion. HGNMI=high-grade non-muscle invasion. HGMI=high-grade muscle invasion. PPV=positive predictive value. NPV=negative predictive value. CI=confidence interval.

**Table S2.** Diagnostic accuracy of the PAIDM and pathologists in validation set 3.

|  | Overall_Accuracy  (95% CI) | LGNMI_Accuracy  (95% CI) | HGNMI_Accuracy  (95% CI) | HGMI_Accuracy  (95% CI) |
| --- | --- | --- | --- | --- |
| PAIDM | 0.793  (0.721-0.865) | 0.842  (0.772-0.912) | 0.848  (0.777-0.919) | 0.848  (0.738-0.957) |
| Junior  pathologist 1 | 0.676  (0.586-0.766) | 0.760  (0.672-0.848) | 0.710  (0.620-0.801) | 0.792  (0.676-0.908) |
| Junior  pathologist 2 | 0.743  (0.667-0.820) | 0.747  (0.666-0.829) | 0.732  (0.645-0.819) | 0.870  (0.771-0.969) |
| Intermediate pathologist 1 | 0.779  (0.703-0.856) | 0.766  (0.683-0.850) | 0.750  (0.671-0.829) | 0.922  (0.844-1) |
| Intermediate pathologist 2 | 0.856  (0.793-0.919) | 0.814  (0.732-0.896) | 0.818  (0.746-0.890) | 1  (1-1) |
| Senior  pathologist 1 | 0.901  (0.856-0.946) | 0.872  (0.805-0.939) | 0.873  (0.806-0.940) | 1  (1-1) |
| Senior  pathologist 2 | 0.910  (0.856-0.964) | 0.908  (0.851-0.965) | 0.905  (0.849-0.961) | 0.953  (0.906 -1) |

PAIDM=pathological artificial intelligence diagnostic model. LGNMI=low-grade non-muscle invasion. HGNMI=high-grade non-muscle invasion. HGMI=high-grade muscle invasion. CI=confidence interval.


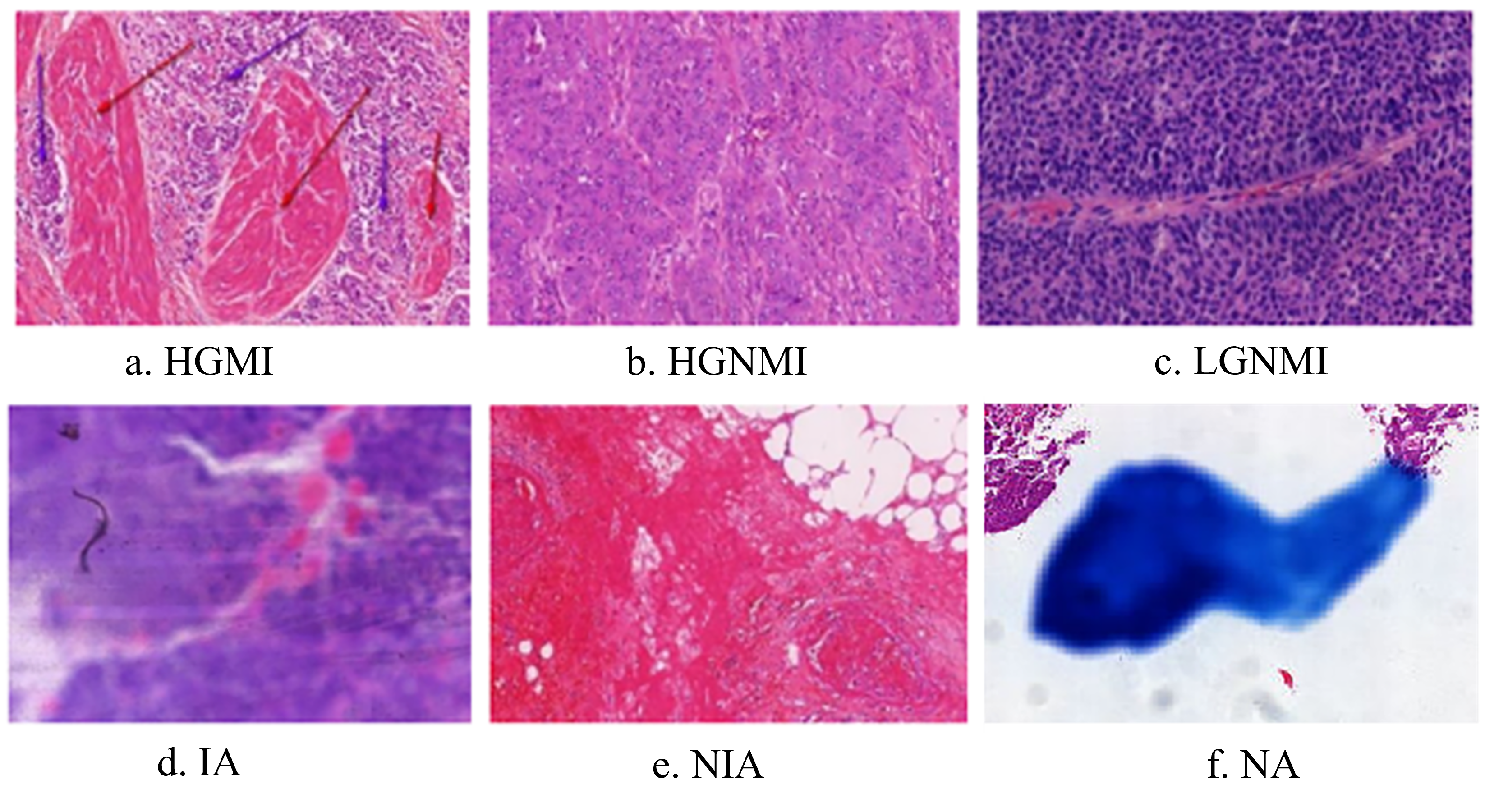


**Figure S1. Examples of six labels for fully annotated images.** (a) HGMI contained both high-grade tumour cells and bladder muscle tissue. (b) HGNMI only contained high-grade tumour cells. (c) LGNMI only contained low-grade tumour cells. (d) IA was defined as the blurred area due to scanning. (e) NIA included all normal mesenchymal cells. (f) NA indicated the noncellular area due to staining. HGMI=high-grade muscle invasion. HGNMI=high-grade non-muscle invasion. LGNMI=low-grade non-muscle invasion. IA=illegible area. NIA=normal interstitial area. NA=noise area.


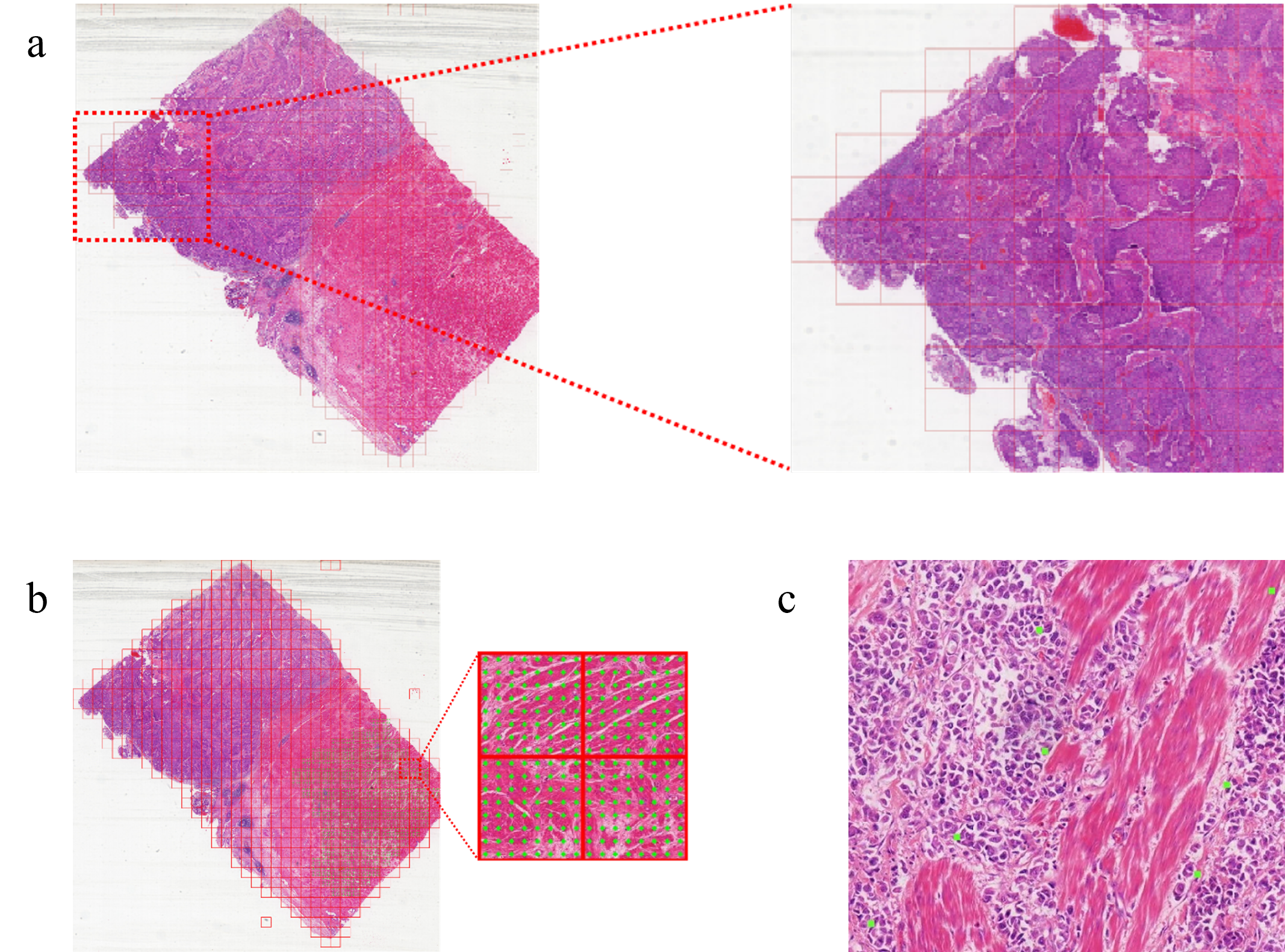


**Figure S2. Image preprocessing and patch sampling.** (a) The OTSU algorithm was used to eliminate the white backgrounds to improve the efficiency of diagnosis and analysis. The red boxes are the preserved tissue area, and the white backgrounds were removed. (b) The sliding window method was used to extract patches of the HGNMI, LGNMI, IA, NIA and NA types. The sampling points were within the annotation region to guarantee that each patch comprised just the certain kind of tissue. (c) The point-based labelling method was adopted to extract the typical patches of HGMI. The sampling points were marked at the junction of tumour tissue and muscle tissue. The green points are the sampling points of the HGMI type. HGMI=high-grade muscle invasion. HGNMI=high-grade non-muscle invasion. LGNMI=low-grade non-muscle invasion. IA=illegible area. NIA=normal interstitial area. NA=noise area.


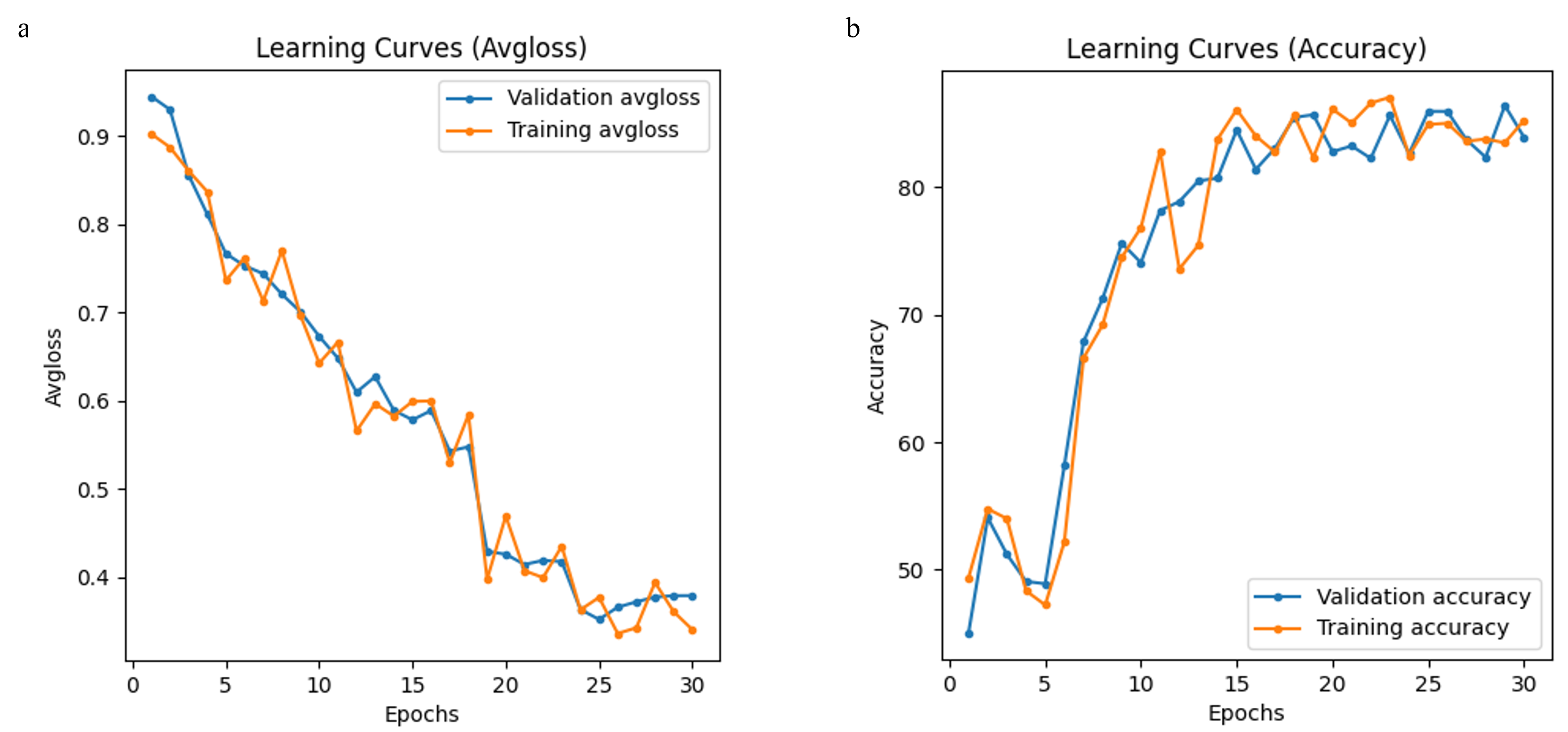


**Figure S3. Learning curves of the PAIDM.** The learning curves of the PAIDM show the gradual decrease of loss (a) and increase of accuracy (b) during the training process. PAIDM=pathological artificial intelligence diagnostic model.


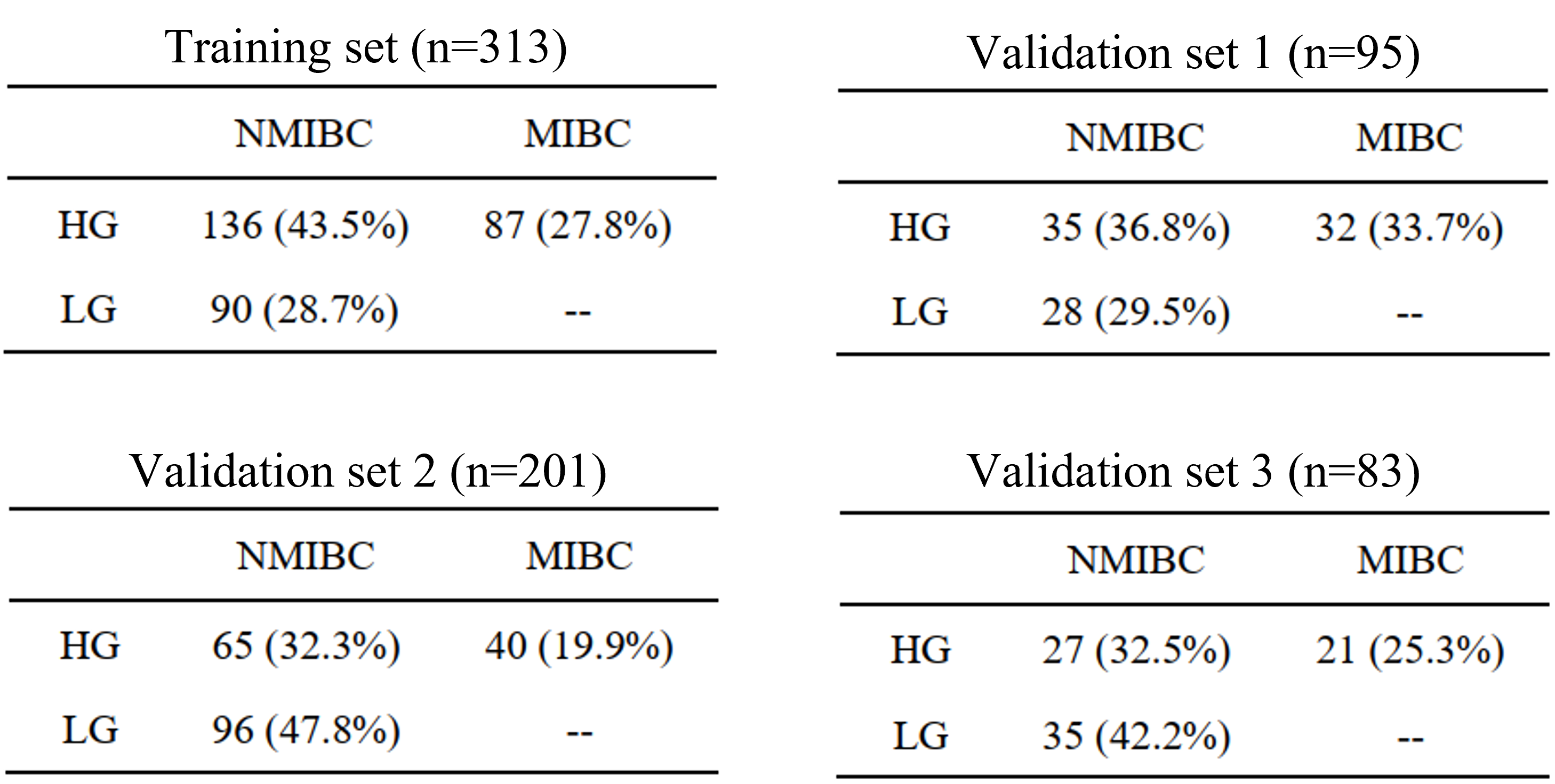


**Figure S4. The proportion of patients with HGMI, HGNMI and LGNMI subtypes in training set and three validation sets.** HG= high grade. LG= low grade. NMIBC=non-muscle-invasive bladder cancer. MIBC= muscle-invasive bladder cancer.


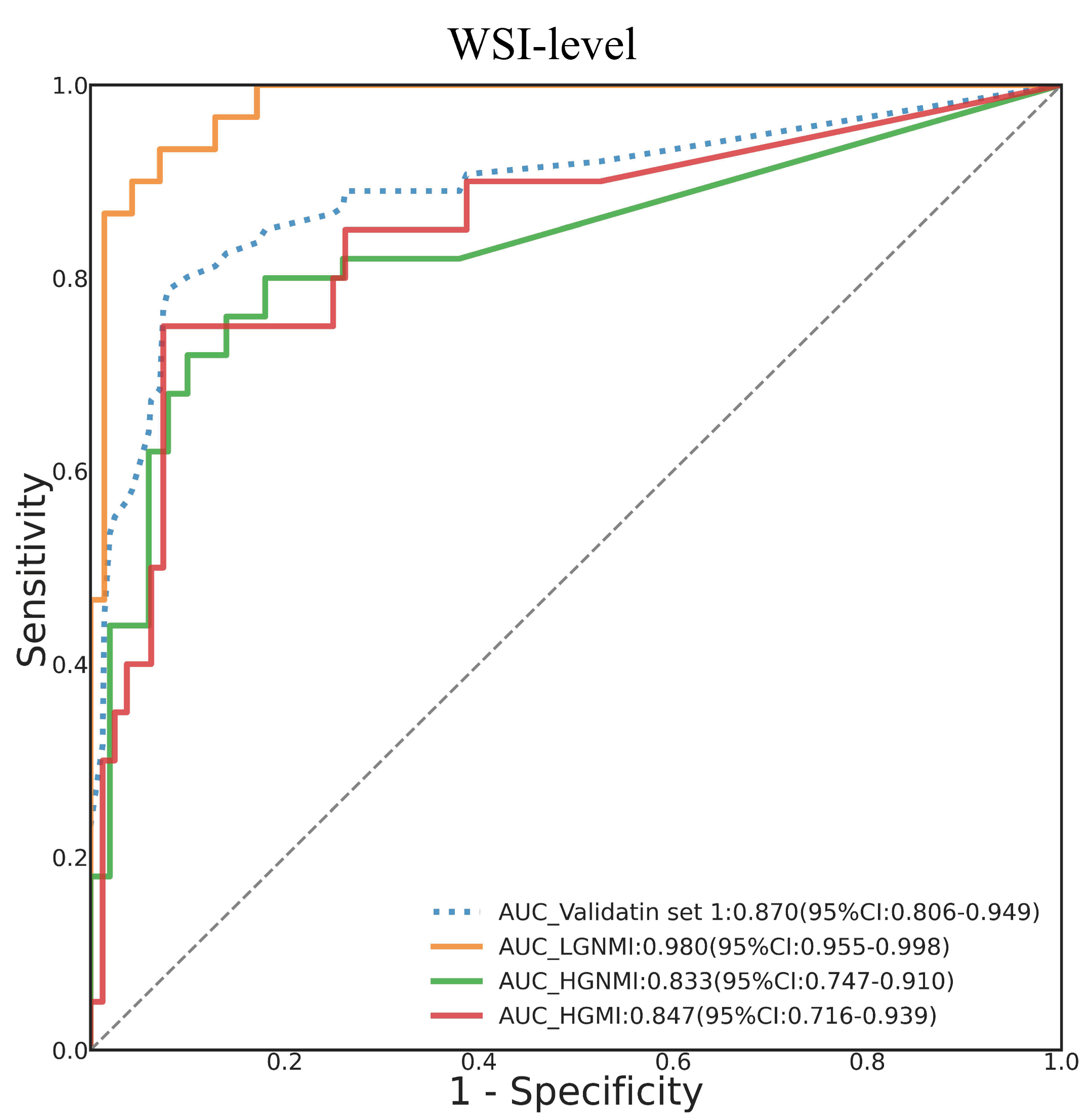


**Figure S5.** **ROC curves for WSI-level diagnostic performance of the PAIDM in validation set 1.** PAIDM=pathological artificial intelligence diagnostic model. WSI= whole slide image. ROC=receiver operating characteristic. AUC=area under the curve. LGNMI=low-grade non-muscle invasion. HGNMI=high-grade non-muscle invasion. HGMI=high-grade muscle invasion.


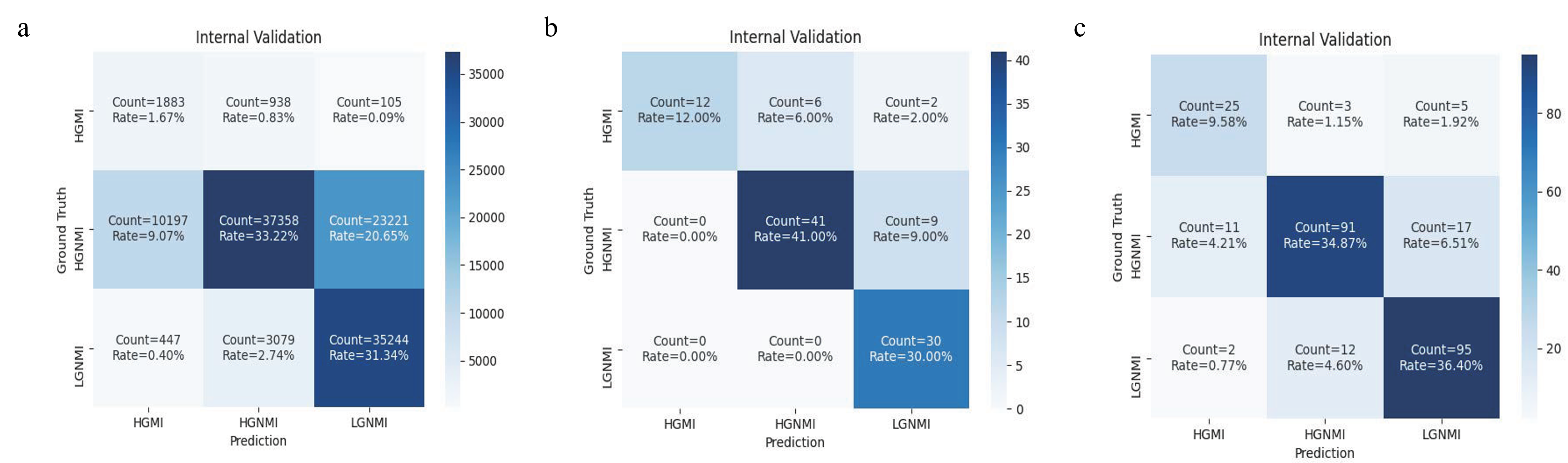


**Figure S6. Confusion matrices of the PAIDM in the two validation sets.** (a) Confusion matrix for the patch-level classification in validation set 1. (b) Confusion matrix for the WSI-level classification in validation set 1. (c) Confusion matrix for the WSI-level classification in validation set 2. PAIDM=pathological artificial intelligence diagnostic model. WSI= whole slide image. LGNMI=low-grade non-muscle invasion. HGNMI=high-grade non-muscle invasion. HGMI=high-grade muscle invasion.


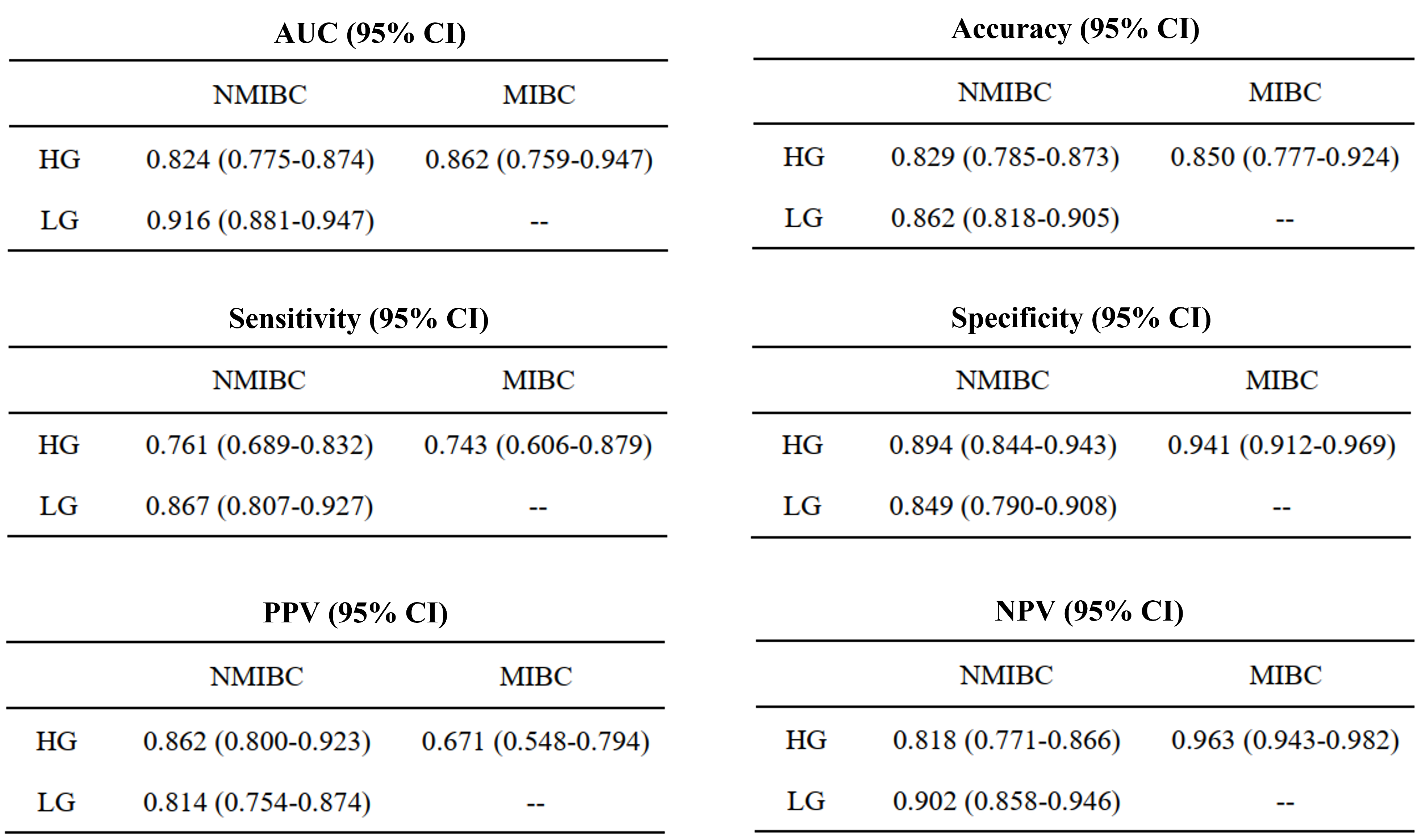


**Figure S7. Diagnostic parameters to assess the PAIDM at the WSI level in validation set 2.** AUC=area under the curve. CI=confidence interval. PPV=positive predictive value. NPV=negative predictive value. HG= high grade. LG= low grade. NMIBC=non-muscle-invasive bladder cancer. MIBC= muscle-invasive bladder cancer. PAIDM=pathological artificial intelligence diagnostic model. WSI=whole slide image.


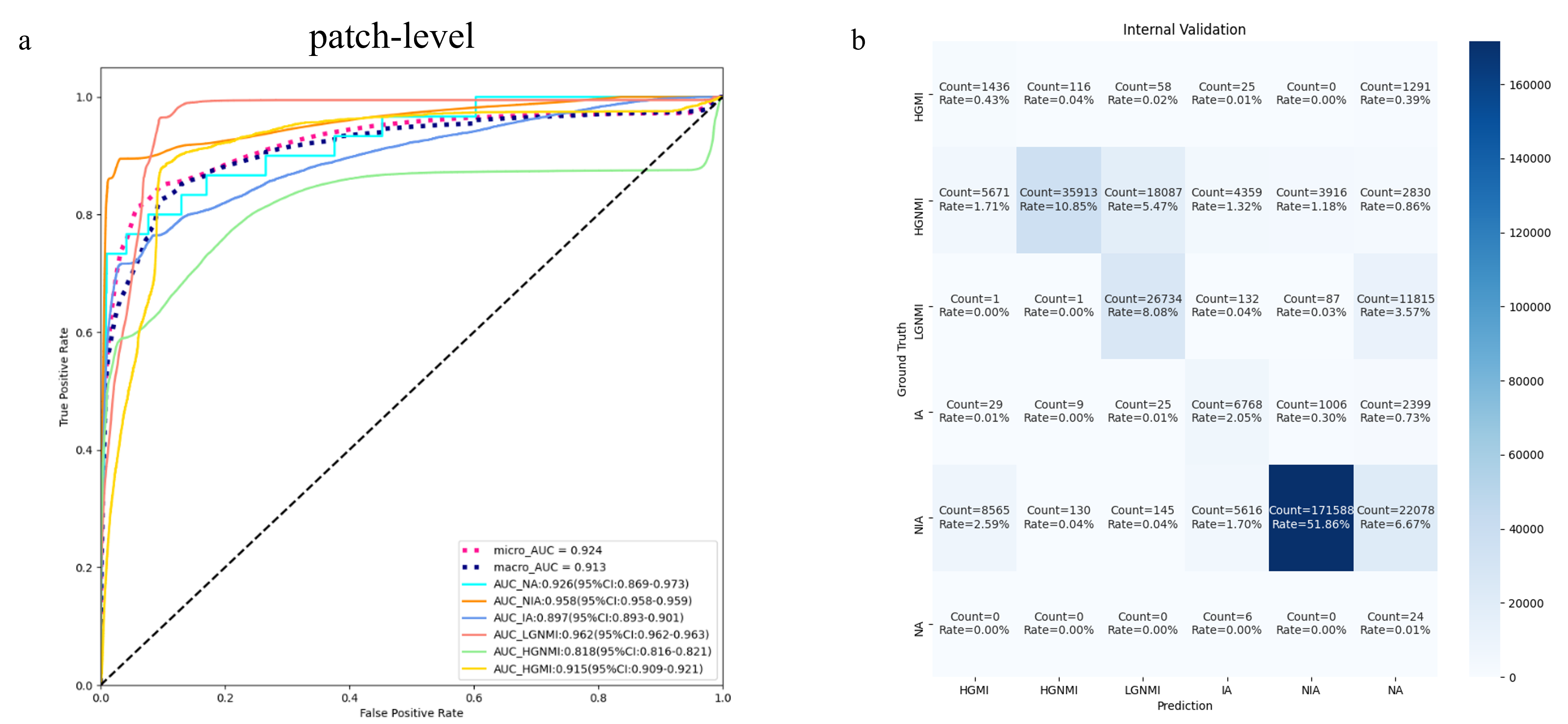


**Figure S8.** **Diagnostic performance of the PAIDM in six-class recognition at the patch level in validation set 1.** (a) ROC curves for patch-level diagnostic performance of six classes. (b) Confusion matrix for patch-level diagnostic performance of six classes. PAIDM=pathological artificial intelligence diagnostic model. ROC=receiver operating characteristic. AUC=area under the curve. HGMI=high-grade muscle invasion. HGNMI=high-grade non-muscle invasion. LGNMI=low-grade non-muscle invasion. IA=illegible area. NIA=normal interstitial area. NA=noise area.
